# Participatory learning design and student well-being in Sino-foreign higher education: engagement and inclusive climate as psychological pathways

**DOI:** 10.3389/fpsyg.2026.1913298

**Published:** 2026-08-26

**Authors:** Zening Zhang

**Affiliations:** School of Marxism, Beijing Foreign Studies University, Beijing, China

**Keywords:** inclusive learning climate, intercultural learning, participatory learning design, Sino-foreign higher education, student engagement, student well-being

## Abstract

**Background:**

Sino-foreign higher education requires students to coordinate academic participation, multilingual communication, intercultural adjustment, and peer collaboration. This study examined whether creative and participatory learning design was associated with student well-being, collaborative engagement, and intercultural learning, and whether student engagement and inclusive learning climate functioned as psychological pathway variables.

**Methods:**

Survey data were collected from 373 undergraduates enrolled in Sino-foreign cooperative programs, followed by semi-structured interviews with 10 students to contextualize the quantitative results. Partial least squares structural equation modeling and bootstrapped mediation analyses were integrated with thematic analysis of the interviews.

**Results:**

Creative and participatory learning design was positively associated with student engagement, inclusive learning climate, student well-being, collaborative engagement, and intercultural learning. Student engagement and inclusive learning climate statistically accounted for part of the associations between learning design and all three outcomes. Interview narratives provided context suggesting that psychological safety, affective support, language negotiation, and intercultural meaning-making may qualify the observed associations.

**Conclusion:**

Participatory learning design appears to be a psychologically relevant correlate of students’ experiences in transnational classrooms. Inclusive, dialogic, and participatory pedagogies warrant longitudinal and intervention-based evaluation; the present cross-sectional findings should be interpreted as associations rather than causal effects.

## 1 Introduction

Sino-foreign cooperative education programs in China are psychologically complex learning settings. Students are expected not only to master disciplinary knowledge, but also to participate across languages, negotiate cultural norms, and build collaborative relationships with peers and teachers from different educational traditions. These demands make Sino-foreign classrooms a critical context for examining how learning environments shape students’ socio-cultural adaptation. Theoretically, the selected outcomes—student well-being, collaborative engagement, and intercultural learning—are privileged over general academic achievement in this study because they align with the three core dimensions of adaptation required in transnational educational settings: the affective, behavioral, and cognitive-cultural dimensions. Well-being represents the affective resolution of psychological demands; collaborative engagement captures the behavioral enactment of cross-cultural teamwork; and intercultural learning reflects the cognitive expansion necessary to navigate differing educational norms.

From an educational psychology perspective, creative and participatory learning design may function as an environmental resource. Open-ended tasks, student voice, reflective dialogue, and collaborative problem-solving can increase perceived autonomy, relatedness (Deci and Ryan, 2000), psychological safety (Edmondson, 1999), and opportunities for social participation. In multilingual and intercultural classrooms, these design features may be especially important because students’ willingness to speak, collaborate, and take intellectual risks is closely tied to affective security and classroom inclusion.

Drawing on third-space theory and social constructivism, this study conceptualizes Sino-foreign classrooms as negotiated psychological and cultural spaces rather than as simple combinations of Chinese and foreign educational systems (Gutiérrez-Santiuste and Ritacco-Real, 2023). Social constructivism emphasizes that learning is socially mediated, while third-space theory highlights the negotiation of meaning, identity, and participation across cultural boundaries. On this basis, inclusive learning climate and student engagement were modeled as theoretically plausible pathway variables. An inclusive climate represents students’ perceptions of recognition, support, and psychological safety, whereas engagement represents their behavioral, emotional, and cognitive participation in classroom activity. The proposed ordering is therefore a theory-guided statistical model; it is not evidence that these processes necessarily unfold in that temporal sequence.

Recent literature on transnational learning and Collaborative Online International Learning (COIL) has increasingly recognized the importance of intercultural competence and social interaction (Fukkink et al., 2024; Hackett et al., 2023). However, much of this research has examined multicultural environments primarily from macro-institutional perspectives or focused strictly on curriculum transfer. While studies have highlighted the developmental benefits of cross-cultural teamwork (Lee et al., 2025; Shen and De La Garza, 2025), less is known about the micro-level psychological pathways through which specific classroom designs facilitate these outcomes in Sino-foreign contexts.

Concurrently, emerging research in educational psychology emphasizes that teaching and learning environments are associated with students’ socio-emotional skills and well-being (Boman et al., 2025; Wang et al., 2025). Well-being and collaborative engagement are increasingly viewed not merely as byproducts of academic success, but as dimensions of how students adapt and participate in higher education (Castillo, 2025; Fang et al., 2025). However, prior research has more often examined engagement and inclusive climates in domestic or online international-learning settings than in co-located Sino-foreign programs. The present study therefore examines whether participatory pedagogy, engagement, inclusion, and developmental outcomes are statistically related within this specific transnational context, without assuming that classroom design alone determines those outcomes.

### 1.1 Current study aim and hypotheses

The current study aimed to examine how creative and participatory learning design is associated with student well-being, collaborative engagement, and intercultural learning in Sino-foreign higher education, and to test whether student engagement and inclusive learning climate statistically account for part of these associations. Guided by Self-Determination Theory (Deci and Ryan, 2000), psychological safety (Edmondson, 1999), social constructivism, and third-space theory, the study used a sequential explanatory mixed-methods design to test six confirmatory hypotheses and then use interview narratives to clarify the quantitative patterns.

In the integrated model, creative and participatory learning design (CPP) is the learning-environment predictor and encompasses open-ended tasks, student voice, reflective dialogue, collaborative problem-solving, and inclusive interaction. Student engagement captures behavioral, emotional, and cognitive participation, whereas inclusive learning climate captures recognition, support, belonging, and willingness to participate across linguistic and cultural differences. The outcomes represent complementary dimensions of adaptation: student well-being reflects affective functioning in the learning context, collaborative engagement reflects coordinated participation in group tasks, and intercultural learning reflects cultural awareness, perspective-taking, and confidence in intercultural communication.

#### 1.1.1 Direct pedagogical associations

Drawing on environmental-resource perspectives, the model tested whether pedagogical designs incorporating reflective dialogue and open-ended tasks were positively associated with outcomes theoretically related to autonomy and relatedness. Accordingly, the following direct structural paths were hypothesized:

*H1*: Creative and participatory learning design (CPP) is positively associated with (a) student well-being, (b) collaborative engagement, and (c) intercultural learning.

#### 1.1.2 Associations with the pathway variables

Consistent with psychological-safety and social-participation perspectives, the model tested whether perceived participatory design was positively associated with students’ immediate engagement and perceptions of inclusion. Accordingly, the following pathway hypotheses were proposed:

*H2*: CPP is positively associated with (a) student engagement and (b) inclusive learning climate.

*H3*: Student engagement is positively associated with an inclusive learning climate.

#### 1.1.3 Indirect pathways

Aligning with the socially mediated nature of learning, behavioral participation and perceived affective security were examined as statistical correlates that might account for part of the associations between CPP and the developmental outcomes:

*H4*: Student engagement is positively associated with (a) student well-being, (b) collaborative engagement, and (c) intercultural learning.

*H5*: Inclusive learning climate is positively associated with (a) student well-being, (b) collaborative engagement, and (c) intercultural learning.

*H6*: Student engagement and inclusive learning climate will partially mediate the associations between CPP and the three developmental outcomes (simple mediation).

In addition to these theoretically driven confirmatory hypotheses, two exploratory analyses were conducted to deepen the contextual understanding of the cross-sectional data: (1) evaluating a possible sequential association (CPP → student engagement → inclusive learning climate → outcomes), and (2) a descriptive subgroup comparison to illustrate outcome distributions across perceived CPP levels.

## 2 Materials and methods

### 2.1 Study design and psychological model

**Figure 1:**
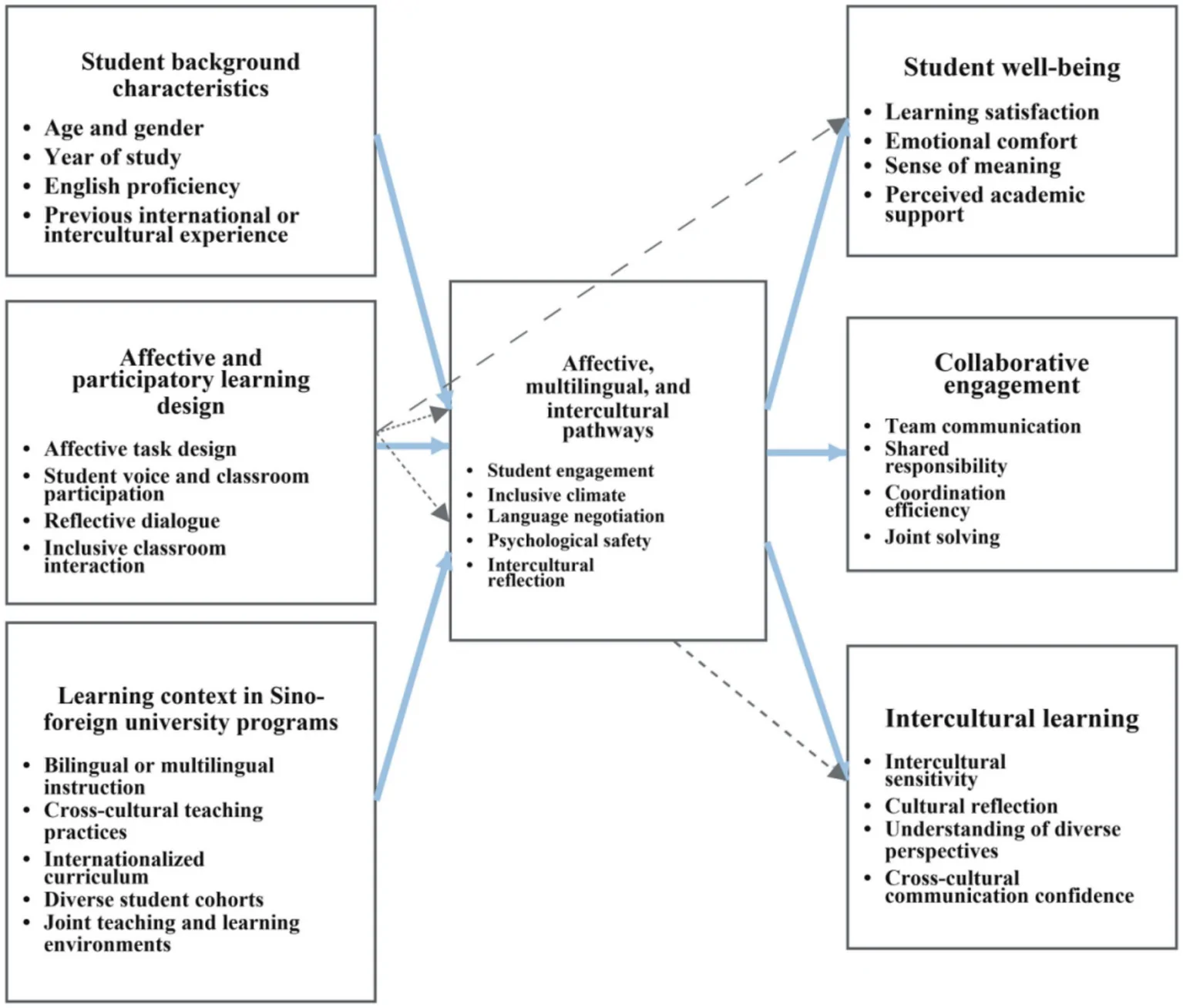
A proposed framework of affective, multilingual, and intercultural pathways to student well-being and collaborative engagement in Sino-foreign higher education. The left column summarizes student background characteristics, affective and participatory learning design, and the Sino-foreign learning context; the central box represents the proposed affective, multilingual, and intercultural pathway variables; and the right column shows the three focal outcomes: student well-being, collaborative engagement, and intercultural learning. Dashed arrows indicate contextual background factors collected for sample description, rather than hypothesized structural paths estimated in the quantitative model.

This study used a sequential explanatory mixed-methods design to examine how students’ perceptions of creative and participatory learning design were related to psychological and developmental outcomes in Sino-foreign higher education. The quantitative phase tested a structural model linking learning design with student well-being, collaborative engagement, and intercultural learning. The qualitative phase used interview narratives to clarify how students experienced psychological safety, language negotiation, classroom inclusion, and collaborative participation (see Figure 1).

Student engagement and inclusive learning climate were specified as psychological pathway variables. Student engagement captured students’ behavioral, emotional, and cognitive participation in learning (Fredricks et al., 2004; Kuh, 2009), whereas inclusive learning climate captured perceived recognition, support, and belonging in a multilingual classroom. This specification allowed the analysis to move beyond direct associations and examine how pedagogical environments may become psychologically meaningful for students.

**Figure 2:**
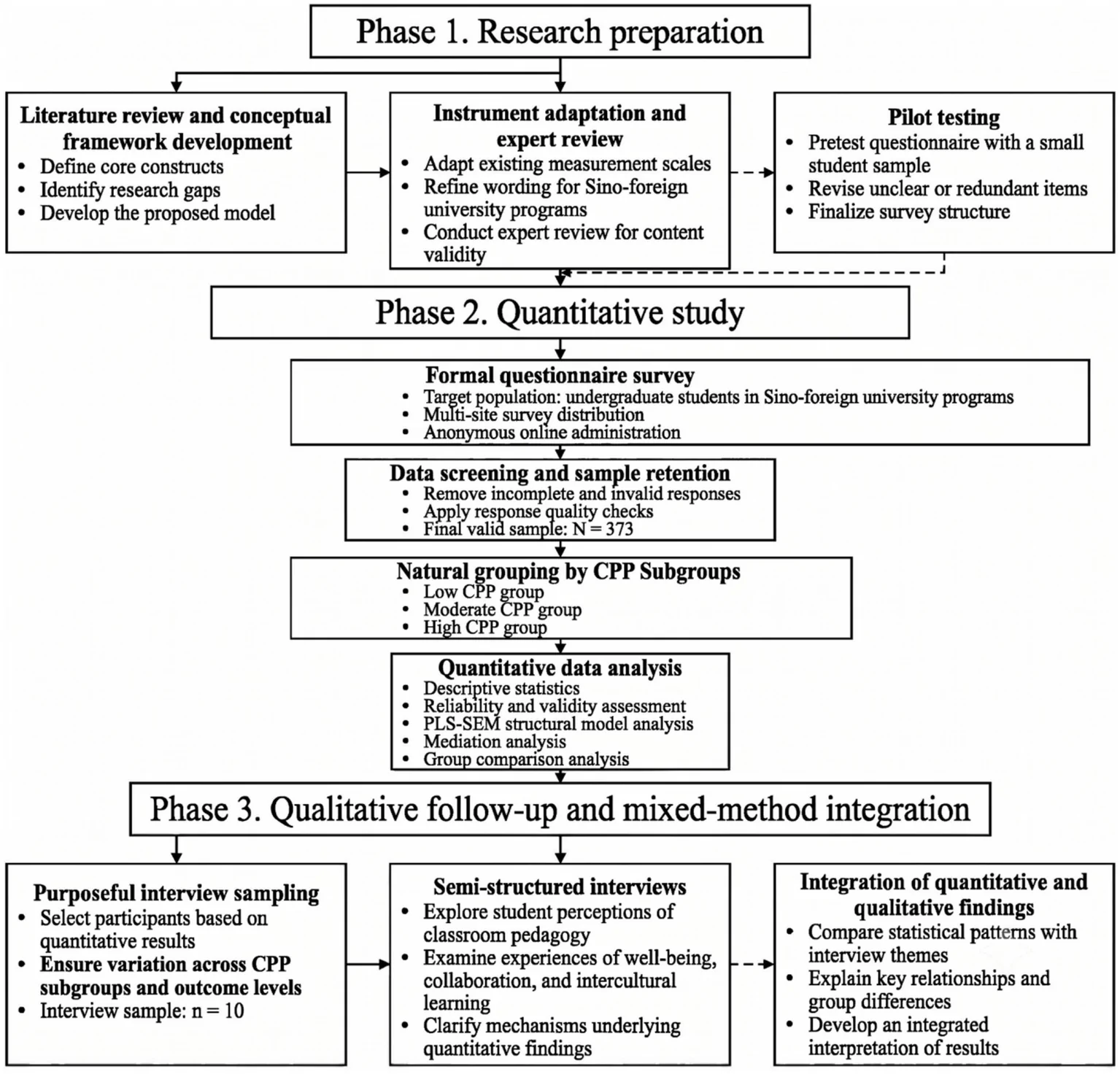
Research design and mixed-method data collection procedures. Phase 1 shows literature review, conceptual framework development, instrument adaptation, expert review, and pilot testing. Phase 2 shows the quantitative survey, data screening, final valid sample retention (*N* = 373), natural grouping by perceived CPP subgroups, and quantitative analyses. Phase 3 shows purposeful interview sampling (*n* = 10), semi-structured interviews, and integration of quantitative and qualitative findings.

The study proceeded in three stages. First, relevant literature on student well-being, engagement, collaborative learning, and intercultural development was reviewed to identify the psychological constructs to be measured. Second, a structured questionnaire was adapted and pilot tested for the Sino-foreign classroom context. Third, quantitative findings were followed by interviews so that statistical patterns could be interpreted in relation to students’ lived learning experiences (see Figure 2).

### 2.2 Participants and educational setting

Participants were undergraduate students enrolled in Sino-foreign cooperative programs in mainland China. These programs typically involve bilingual or multilingual instruction, imported or jointly developed curricula, cross-cultural classroom interaction, and instruction by Chinese and foreign teachers. Psychologically, this context requires students to regulate academic expectations, communicate across languages, and form collaborative relationships in a culturally mixed learning environment.

Undergraduates were selected because this population is developmentally relevant for research on academic adaptation, identity formation, peer belonging, and socio-emotional learning. Sino-foreign programs also provide a natural setting in which students’ well-being, classroom engagement, and intercultural learning can be examined in relation to everyday instructional practices.

To capture variation in the transnational educational context, data were collected from four Sino-foreign cooperative university programs in eastern and northern China. To maintain strict institutional confidentiality and mitigate evaluation apprehension among student respondents, the specific names of these universities are withheld. The programs included partnerships with British, American, and Australian institutions and represented variation in disciplinary orientation, language exposure, and classroom organization.

After data screening, 373 valid responses were retained for quantitative analysis. The sample included students from all four undergraduate years and from several disciplinary areas, including business and management, engineering and technology, social sciences and humanities, arts and media, and other applied fields. Ten students were subsequently selected for follow-up interviews to provide psychologically rich explanations of the survey patterns.

All participants were adults aged 18 years or older and were enrolled across the four undergraduate years (see Table 1).

**Table 1:** Demographic characteristics of participants in Sino-Foreign university programs.

Characteristic	Category	*n*	%
Gender	Male	161	43.2
	Female	212	56.8
Year of study	First year	89	23.9
	Second year	104	27.9
	Third year	96	25.7
	Fourth year	84	22.5
Major field	Business and management	118	31.6
	Engineering and technology	91	24.4
	Social sciences and humanities	84	22.5
	Arts and media	43	11.5
	Other disciplines	37	9.9
English proficiency	CET-4 or below	126	33.8
	CET-6	167	44.8
	IELTS 6.0/equivalent or above	80	21.4
Prior international or intercultural experience	None	149	39.9
	Short-term exposure	154	41.3
	Sustained experience	70	18.8
CPP subgroups	Low	109	29.2
	Moderate	149	39.9
	High	115	30.8

### 2.3 Instruments and psychological measures

A structured self-report questionnaire was used to measure students’ perceptions of classroom design, psychological pathway variables, and developmental outcomes. Items were adapted from established higher education and collaborative learning instruments (National Survey of Student Engagement [NSSE], 2021; Schaufeli et al., 2006). To ensure cross-cultural semantic equivalence, the original English items underwent a rigorous forward- and back-translation procedure conducted by two bilingual researchers. The translated pool of items was then reviewed for contextual fit with Sino-foreign cooperative classrooms, emphasizing psychological clarity, construct coverage, and linguistic comprehensibility.

The questionnaire included measures of creative and participatory learning design, student engagement, inclusive learning climate, student well-being, collaborative engagement, and intercultural learning. Although creative and participatory learning design (CPP) conceptually encompasses five sub-dimensions, it was analytically specified as a unidimensional, first-order reflective construct in this study. This specification decision was driven by the high inter-correlations among its 20 indicators—which collectively reflect a holistic pedagogical environment rather than competing instructional domains—and the necessity for structural parsimony, ensuring that the primary predictive pathways remained interpretable without the unnecessary complexity of a higher-order hierarchical model. The constructs were treated as reflective latent variables because the items were conceptualized as observable indicators of broader psychological experiences. While several subdimensions (e.g., within student well-being and intercultural learning) were operationalized using two high-loading indicators, this structural decision was deliberately made to minimize respondent fatigue within a comprehensive survey battery. Psychometrically, two-item measures are considered acceptable and capable of producing stable latent variables when the items are highly correlated and theoretically narrow in scope (Eisingerich and Rubera, 2010; Hayduk and Littvay, 2012). The subsequent measurement model evaluation confirmed the strong internal consistency and construct representation of these dimensions. Student engagement was represented by six items covering behavioral, emotional, and cognitive participation, whereas inclusive learning climate was represented by five items addressing recognition, support, belonging, and willingness to participate across linguistic and cultural differences.

All items used a five-point Likert-type response scale ranging from 1 (strongly disagree) to 5 (strongly agree). Higher scores indicated stronger endorsement of the relevant learning experience or psychological outcome. A five-point scale was selected to reduce response burden and support comprehension in an online multilingual learning context.

To strengthen content validity and linguistic accuracy, an expert panel consisting of five researchers and senior instructors with extensive international teaching experience — including two independent scholars holding PhDs in educational psychology and one specialist in quantitative psychometrics — reviewed the preliminary questionnaire. Their feedback focused on whether the items captured the intended psychological constructs and whether wording was appropriate for Sino-foreign students, leading to minor phrasing modifications to prevent cultural ambiguity. Subsequently, a pilot study was conducted with a small sub-sample of target students (*n* = 45). The pilot-testing statistics demonstrated acceptable preliminary internal consistency, with Cronbach’s alpha coefficients exceeding 0.75 for all tentative subscales, confirming the feasibility of the instrument before formal administration (see Table 2).

**Table 2:** Measurement items and constructs of creative and participatory learning design, student engagement, inclusive learning climate, student well-being, collaborative engagement, and intercultural learning.

Construct	Dimension	Item code(s)	No. of items	Sample item(s)/measured content	Response format
Creative and participatory pedagogies	Creative task design	CP1–CP4	4	“The course used open-ended tasks that encouraged original thinking.”	5-point Likert (1–5)
	Student voice and participation	CP5–CP8	4	“I was encouraged to express my own ideas and perspectives in class.”	5-point Likert (1–5)
	Reflective dialogue	CP9–CP12	4	“Class activities encouraged me to reflect critically on my learning experiences.”	5-point Likert (1–5)
	Collaborative problem-solving	CP13–CP16	4	“The course required students to work together to solve complex problems.”	5-point Likert (1–5)
	Inclusive classroom interaction	CP17–CP20	4	“Students from different backgrounds were given equal opportunities to participate.”	5-point Likert (1–5)
Student engagement	Behavioral, emotional, and cognitive participation	SE1-SE6	6	Items assessed active participation, emotional involvement, and cognitive investment in learning activities.	5-point Likert (1–5)
Inclusive learning climate	Recognition, support, belonging, and willingness to participate	ILC1-ILC5	5	Items assessed whether students felt recognized, supported, included, and able to participate across linguistic and cultural differences.	5-point Likert (1–5)
Student well-being	Learning satisfaction	SWB1–SWB2	2	“I am satisfied with my learning experience in this program.”	5-point Likert (1–5)
	Emotional comfort	SWB3–SWB4	2	“I generally feel emotionally comfortable during class participation.”	5-point Likert (1–5)
	Sense of meaning	SWB5–SWB6	2	“My learning in this program feels meaningful to me.”	5-point Likert (1–5)
	Perceived academic support	SWB7–SWB8	2	“I feel supported by teachers and peers in my learning.”	5-point Likert (1–5)
Collaborative engagement	Team communication	COL1–COL2	2	“My group communicated effectively when completing shared tasks.”	5-point Likert (1–5)
	Shared responsibility	COL3–COL4	2	“Group members shared responsibilities fairly.”	5-point Likert (1–5)
	Coordination efficiency	COL5–COL6	2	“Our team coordinated well to complete course assignments.”	5-point Likert (1–5)
	Joint problem-solving	COL7–COL8	2	“My group was able to solve problems collaboratively.”	5-point Likert (1–5)
Intercultural learning	Intercultural sensitivity	IL1–IL2	2	“I have become more sensitive to cultural differences through this program.”	5-point Likert (1–5)
	Cultural reflection	IL3–IL4	2	“This learning experience encouraged me to reflect on my own cultural assumptions.”	5-point Likert (1–5)
	Understanding diverse perspectives	IL5–IL6	2	“I learned to understand issues from perspectives different from my own.”	5-point Likert (1–5)
	Cross-cultural communication confidence	IL7–IL8	2	“I feel more confident communicating with people from different cultural backgrounds.”	5-point Likert (1–5)

CPP, creative and participatory pedagogies; SE, student engagement; ILC, inclusive learning climate; SWB, student well-being; CE, collaborative engagement; IL, intercultural learning. All items were rated on a five-point Likert-type scale from 1 (strongly disagree) to 5 (strongly agree).

### 2.4 Procedure

Data collection commenced in October 2025, after ethical approval had been granted, and proceeded in three stages: pilot testing, formal survey administration, and follow-up interviews. The pilot stage examined whether students understood the items as intended and whether any wording needed refinement. The formal survey was then distributed online, followed by interviews designed to elicit students’ subjective experiences of participation, support, collaboration, and intercultural adjustment.

Before recruitment, permission to distribute the study invitation was obtained from administrators of all four participating Sino-foreign cooperative programs. The formal survey was administered anonymously online to undergraduate students. Questionnaires were distributed through course channels, student project groups, and teaching management platforms to broaden access while reducing pressure to participate.

A total of 450 survey invitations were distributed, yielding 412 returned responses (an approximate response rate of 91.6%). During data screening, 39 responses with extensive missing data, abnormal completion times, or repetitive response patterns were excluded using listwise deletion. To ensure sample adequacy, an *a priori* power analysis using G*Power (Faul et al., 2009) was conducted. The results indicated that a minimum sample size of 119 was required to detect a medium effect size ( $f^{2}$ = 0.15) with 95% statistical power and an alpha level of 0.05. Therefore, the final analytical sample consisting of 373 valid questionnaires provided robust statistical power for the subsequent structural equation modeling. Because the study was observational and based on self-reported perceptions, the findings are interpreted as associations rather than experimental effects.

After the quantitative phase, ten undergraduate students were selected for semi-structured interviews through maximum variation purposive sampling based on their quantitative CPP scores and outcome levels. The interview protocol was developed based on the quantitative findings and existing literature, focusing on students’ lived experiences of classroom inclusion, peer collaboration, language negotiation, and emotional well-being. Each participant completed one in-depth, semi-structured interview lasting between 45 and 65 min, conducted either face-to-face or via secure video conferencing. All interviews were audio-recorded, anonymized, and transcribed verbatim, yielding approximately 130 pages of text. No new codes, thematic properties, or conceptual dimensions were identified in the final two interviews; this pattern indicated sufficient information power for the explanatory purpose of the present dataset, while not implying population-level representativeness.

### 2.5 Ethical considerations

This study involved anonymous survey responses and follow-up interview data from undergraduate students. The study was reviewed and approved by the Institutional Review Board of the School of Marxism, Beijing Foreign Studies University (Approval No. 22JZD023; approved 5 October 2025). Before participation, students received written information about the study purpose, the voluntary nature of participation, confidentiality protections, their right to decline questions or withdraw without penalty, and their option to request deletion of collected data, and they provided informed consent. All participants were aged 18 years or older; therefore, parental or guardian consent was not required. All procedures complied with institutional and national ethical standards for research involving human participants.

### 2.6 Statistical and qualitative analysis strategy

Data analysis proceeded through descriptive statistics, measurement-model evaluation, structural-model estimation, mediation analysis, supplementary subgroup comparison, and qualitative thematic interpretation. Partial Least Squares Structural Equation Modeling (PLS-SEM) was chosen as the primary analytical approach and was conducted using SmartPLS 4 software (Ringle et al., 2022). PLS-SEM was deemed more appropriate than covariance-based SEM (CB-SEM) for this study due to its robustness with non-normally distributed data, its suitability for complex models incorporating multiple mediation pathways, and its focus on variance explanation and predictive capability rather than global model fit (Hair et al., 2019). To maintain model parsimony and ensure that statistical power was concentrated on testing the hypothesized psychological pathways, student background characteristics (e.g., age, gender, English proficiency) were utilized strictly for sample contextualization (as reported in Table 1) and were excluded from the structural model estimation as control covariates. Parameter estimates and standard errors were generated using the standard PLS algorithm alongside a bias-corrected and accelerated (BCa) bootstrapping procedure with 5,000 resamples.

Reliability and validity were assessed using standardized item loadings, Cronbach’s alpha, composite reliability, and average variance extracted. These indicators evaluated internal consistency and convergent validity for each latent construct. Discriminant validity was assessed using both the traditional Fornell-Larcker criterion and the more robust Heterotrait-Monotrait (HTMT) ratio of correlations (Henseler et al., 2015) to confirm that the psychological constructs represented related but empirically distinct dimensions of student experience. Furthermore, before examining the structural paths, multicollinearity among the constructs was evaluated using the variance inflation factor (VIF) to ensure that the structural estimates were not compromised by highly correlated predictors.

In addition to structural and mediation analyses, a supplementary subgroup analysis stratified students into low, moderate, and high groups using tertile cut-off points for the CPP composite score. The grouping variable was therefore the primary predictor rather than the student-engagement mediator. This analysis was intended only to translate the continuous association into an accessible descriptive display of outcome distributions across perceived CPP levels. Because the groups were created from the predictor itself, the subgroup comparisons were not treated as independent confirmation of the structural model or as evidence of a pedagogical intervention effect.

Interview data were analyzed by the sole author using inductive-deductive thematic analysis following the six-phase framework of Braun and Clarke (2006). Transcripts were managed and coded in NVivo 14. Initial open codes were grouped into descriptive codes and then synthesized into interpretive themes. To support trustworthiness, transcripts and preliminary themes were returned to three participants for member checking; the author also conducted peer debriefing with colleagues at the author’s institution and maintained a reflexive journal and an audit trail. Because all transcripts were coded by the sole author, inter-coder reliability was not calculated. This single-coder design is acknowledged as a limitation. As a native Chinese researcher teaching within a Sino-foreign institution, the author practiced reflexivity by bracketing personal pedagogical assumptions throughout the analysis. Qualitative themes were used to interpret and contextualize the quantitative findings, not to serve as independent causal proof.

## 3 Results

### 3.1 Preliminary descriptive results

Descriptive statistics indicated that students generally reported moderately high levels of perceived participatory learning design, inclusive learning climate, and collaborative engagement. From a psychological perspective, this suggests that many respondents experienced the Sino-foreign classroom as socially interactive and supportive, although variability remained across students.

Correlation analysis showed positive associations among creative and participatory learning design, student engagement, inclusive learning climate, student well-being, collaborative engagement, and intercultural learning. These associations were consistent with the assumption that classroom design, psychological participation, and socio-emotional outcomes are interconnected in transnational learning environments (see Table 3).

**Table 3:** Descriptive statistics and correlations among the main study variables.

Variable	*M*	*SD*	Skewness	Kurtosis	1	2	3	4	5	6
1. Creative and participatory pedagogies	3.87	0.56	−0.42	0.31	1					
2. Student engagement	3.79	0.58	−0.35	0.18	0.61**	1				
3. Inclusive learning climate	3.91	0.54	−0.47	0.42	0.64**	0.58**	1			
4. Student well-being	3.74	0.6	−0.28	0.09	0.55**	0.62**	0.57**	1		
5. Collaborative engagement	3.81	0.57	−0.31	0.14	0.59**	0.54**	0.49**	0.51**	1	
6. Intercultural learning	3.69	0.62	−0.22	−0.06	0.47**	0.45**	0.52**	0.48**	0.56**	1

*N* = 373. ** *p* < 0.01 (two-tailed).

### 3.2 Measurement model evaluation

Before testing the structural model, the measurement model was evaluated to ensure that the psychological constructs could be interpreted with acceptable reliability and validity. All latent constructs were specified as reflective constructs because the items were conceptualized as manifestations of the underlying student experience.

The validation results indicated that creative and participatory learning design, student engagement, inclusive learning climate, student well-being, collaborative engagement, and intercultural learning met commonly used psychometric criteria. All constructs satisfied the Fornell-Larcker criterion, and all HTMT ratios were below 0.85 (range = 0.48–0.76), supporting empirical distinction among the latent variables despite their positive correlations. Given the cross-sectional, self-reported design, common method bias was examined using Harman’s single-factor test and full-collinearity VIFs. The largest unrotated component accounted for 36.4% of the variance, and inner VIFs ranged from 1.45 to 2.58, below the 3.3 guideline (Kock, 2015). These diagnostics did not identify a dominant common factor or problematic collinearity; however, they cannot exclude shared-method covariance arising from a single respondent and measurement occasion (see Tables 4, 5).

**Table 4:** Reliability and validity evaluation of the measurement model.

Construct	Item	Loading	Cronbach’s *α*	*CR*	*AVE*
Creative and participatory pedagogies	CP1	0.76	0.93	0.94	0.56
	CP2	0.74			
	CP3	0.79			
	CP4	0.77			
	CP5	0.72			
	CP6	0.75			
	CP7	0.78			
	CP8	0.73			
	CP9	0.76			
	CP10	0.81			
	CP11	0.77			
	CP12	0.74			
	CP13	0.75			
	CP14	0.79			
	CP15	0.8			
	CP16	0.76			
	CP17	0.71			
	CP18	0.74			
	CP19	0.78			
	CP20	0.75			
Student engagement	SE1	0.79	0.89	0.92	0.65
	SE2	0.82			
	SE3	0.77			
	SE4	0.81			
	SE5	0.84			
	SE6	0.79			
Inclusive learning climate	ILC1	0.8	0.87	0.91	0.67
	ILC2	0.83			
	ILC3	0.79			
	ILC4	0.84			
	ILC5	0.82			
Student well-being	SWB1	0.78	0.88	0.91	0.62
	SWB2	0.8			
	SWB3	0.77			
	SWB4	0.81			
	SWB5	0.79			
	SWB6	0.8			
	SWB7	0.76			
	SWB8	0.79			
Collaborative engagement	COL1	0.81	0.86	0.9	0.64
	COL2	0.79			
	COL3	0.77			
	COL4	0.82			
	COL5	0.8			
	COL6	0.78			
	COL7	0.79			
	COL8	0.81			
Intercultural learning	IL1	0.77	0.87	0.91	0.63
	IL2	0.8			
	IL3	0.79			
	IL4	0.82			
	IL5	0.78			
	IL6	0.81			
	IL7	0.8			
	IL8	0.77			

**Table 5:** Discriminant validity results (Fornell–Larcker criterion).

Construct	1	2	3	4	5	6
1. CPP	0.748					
2. SE	0.61	0.806				
3. ILC	0.64	0.58	0.819			
4. SWB	0.55	0.62	0.57	0.787		
5. CE	0.59	0.54	0.49	0.51	0.800	
6. IL	0.47	0.45	0.52	0.48	0.56	0.794

### 3.3 Structural model and hypothesis testing

The structural model accounted for 49% of the variance in student well-being, 44% in collaborative engagement, and 38% in intercultural learning. Within this sample, these *R*^2^ values indicate moderate explanatory capacity (Hair et al., 2019). Blindfolding produced positive *Q*^2^ values of 0.32, 0.28, and 0.21, respectively, indicating in-sample predictive relevance rather than verified out-of-sample predictive accuracy. The SRMR value was 0.065, below the commonly reported descriptive guideline of 0.08. Path-specific *f*^2^ values ranged from 0.08 to 0.27, indicating that the statistically significant associations varied from small to moderate in magnitude and should not be evaluated from *p*-values alone.

**Figure 3:**
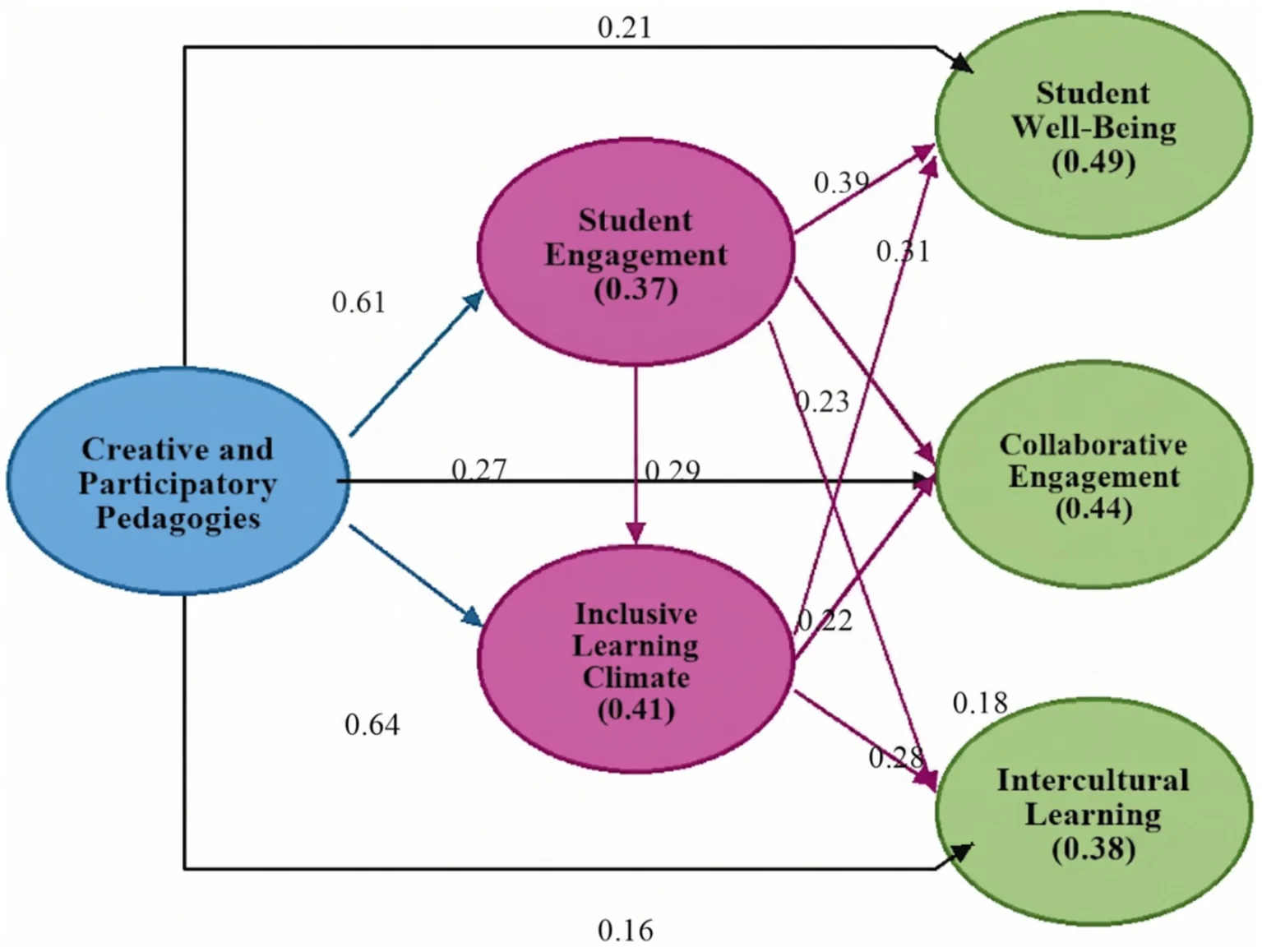
Structural model linking creative and participatory learning design, mediating pathway variables, and student developmental outcomes. The blue oval represents the pedagogical predictor, purple ovals represent student engagement and inclusive learning climate as pathway variables, and green ovals represent student well-being, collaborative engagement, and intercultural learning as outcomes. Arrows show standardized path coefficients; values in parentheses indicate explained variance. All path coefficients shown are statistically significant (*p* < 0.05).

The results suggest that creative and participatory learning design was positively associated with both student engagement and inclusive learning climate. In psychological terms, pedagogical environments that encourage voice, dialogue, and shared problem-solving appear to be related to students’ active participation and sense of recognition within the classroom (see Figure 3).

Regarding direct structural paths, creative and participatory learning design showed significant positive associations with student well-being, collaborative engagement, and intercultural learning. The largest coefficient was observed for collaborative engagement. This pattern is compatible with the conceptual proximity between participatory tasks and peer communication, shared responsibility, and joint task completion, but it does not demonstrate that the design caused those collaborative experiences.

The model also showed positive associations involving student engagement and inclusive learning climate. Student engagement was related to all three outcomes, with the largest coefficient observed for well-being. Inclusive learning climate was likewise related to the outcomes, with comparatively similar coefficients for well-being and intercultural learning. These coefficients describe differentiated correlational patterns; they do not establish that engagement or inclusion caused changes in the outcomes (see Table 6).

**Table 6:** Structural path model for student well-being, collaborative engagement, and intercultural learning.

Path	*β*	SE	*t*	*p*	95% CI	Decision
Creative and participatory pedagogies → student engagement	0.61	0.05	12.43	<0.001	[0.51, 0.70]	Supported
Creative and participatory pedagogies → inclusive learning climate	0.64	0.05	13.18	<0.001	[0.54, 0.73]	Supported
Student engagement → inclusive learning climate	0.29	0.06	4.92	<0.001	[0.17, 0.40]	Supported
Creative and participatory pedagogies → student well-being	0.21	0.07	3.11	0.002	[0.08, 0.34]	Supported
Creative and participatory pedagogies → collaborative engagement	0.27	0.06	4.28	<0.001	[0.15, 0.40]	Supported
Creative and participatory pedagogies → intercultural learning	0.16	0.07	2.39	0.017	[0.03, 0.29]	Supported
Student engagement → student well-being	0.39	0.06	6.57	<0.001	[0.27, 0.50]	Supported
Student engagement → collaborative engagement	0.23	0.07	3.49	<0.001	[0.10, 0.36]	Supported
Student engagement → intercultural learning	0.18	0.07	2.68	0.008	[0.05, 0.31]	Supported
Inclusive learning climate → student well-being	0.31	0.07	4.67	<0.001	[0.18, 0.44]	Supported
Inclusive learning climate → collaborative engagement	0.22	0.06	3.56	<0.001	[0.10, 0.34]	Supported
Inclusive learning climate → intercultural learning	0.28	0.07	4.09	<0.001	[0.15, 0.41]	Supported

CPP, creative and participatory pedagogies; SE, student engagement; ILC, inclusive learning climate; SWB, student well-being; CE, collaborative engagement; IL, intercultural learning; CI, confidence interval. Path coefficients represent standardized direct effects estimated in the structural model.

### 3.4 Relational patterns across affective, multilingual, and intercultural dimensions

Table 3 reports the Spearman correlations among the main learning-design, pathway, and outcome variables. To preserve the full distributional detail without overloading the main-text figure sequence, the combined violin, scatterplot, density, and correlation matrix has been moved unchanged to Supplementary Figure S1. Overall, creative and participatory learning design, student engagement, inclusive learning climate, student well-being, collaborative engagement, and intercultural learning were positively associated.

For the affective dimension, opportunities for expression and reflective discussion were positively associated with emotional comfort, perceived support, and learning value. Collaborative engagement covaried with team communication, coordination, shared responsibility, and joint problem-solving. Inclusive interaction and reflective dialogue were also associated with cultural reflection, understanding of diverse perspectives, and confidence in cross-cultural communication. Thus, students who reported more opportunities to interact, reflect, negotiate meaning, and participate across languages also tended to report more positive developmental experiences; the correlational data do not establish the direction of these relationships.

### 3.5 Mediation and pathway analysis

To achieve a rigorous mixed-methods integration, a joint display analysis was conducted to map the points of convergence, nuance, and extension between the structural equation modeling results and the interview narratives (Supplementary Table S1). Rather than merely using qualitative data as illustrations, the interview findings were systematically analyzed to provide explanatory context for the statistical mediation patterns and to identify contextual conditions not captured by the survey. Analysis of participant narratives revealed three overarching themes: psychological safety in the third space, collaborative synergy through creative tasks, and multilingual-intercultural identity negotiation. These themes extended the structural equation model by clarifying the lived experiences of engagement, perceived academic support, inclusive classroom interaction, and language negotiation.

While the quantitative model showed a positive association between student engagement and well-being, the interviews added an important boundary condition to this broad pattern. Several participants described the initial movement from passive learning toward reflective dialogue as psychologically demanding, particularly when they were expected to contribute in English. As Student 3, a Business major, explained, “Being asked to debate in English initially made me freeze. It wasn’t until the professor explicitly praised my imperfect but culturally unique examples that I felt safe enough to actually engage.” Rather than demonstrating a statistical moderation effect, these accounts suggest that language confidence, error tolerance, and peer trust may shape how participatory tasks are experienced. They therefore qualify, rather than causally verify, the positive survey associations.

Building on the structural model, this study examined the mediating roles of student engagement and inclusive learning climate, as well as supplementary subgroup differences across perceived CPP subgroups. A bootstrapping procedure with 5,000 resamples estimated 95% bias-corrected confidence intervals for indirect effects. As shown in Table 7, indirect associations between creative and participatory learning design and the three developmental outcomes through student engagement and inclusive learning climate were statistically significant, as none of the confidence intervals included zero.

**Table 7:** Mediation analysis results for affective and inclusive learning pathways via bootstrapping (5,000 resamples).

Hypothesized mediation path	*β*	*SE*	*t*	*p*	95% CI (bias-corrected)	Decision
Simple mediation (student engagement)						
CPP → SE → SWB	0.238	0.045	5.289	< 0.001	[0.155, 0.320]	Supported
CPP → SE → CE	0.140	0.040	3.500	< 0.001	[0.065, 0.215]	Supported
CPP → SE → IL	0.110	0.042	2.619	0.009	[0.030, 0.190]	Supported
Simple mediation (inclusive learning climate)						
CPP → ILC → SWB	0.198	0.046	4.304	< 0.001	[0.110, 0.285]	Supported
CPP → ILC → CE	0.141	0.041	3.439	0.001	[0.062, 0.220]	Supported
CPP → ILC → IL	0.179	0.048	3.729	< 0.001	[0.088, 0.270]	Supported
Sequential mediation						
CPP → SE → ILC → SWB	0.055	0.015	3.667	< 0.001	[0.026, 0.085]	Supported
CPP → SE → ILC → CE	0.039	0.012	3.250	0.001	[0.016, 0.063]	Supported
CPP → SE → ILC → IL	0.050	0.014	3.571	< 0.001	[0.023, 0.078]	Supported

The mediation analysis identified statistically significant indirect associations through student engagement and inclusive learning climate. These estimates are consistent with the proposed pathway interpretation, but they represent a decomposition of contemporaneous covariance. They should not be interpreted as evidence that CPP temporally produced engagement or inclusion and thereby changed student outcomes.

To contextualize the structural findings, the sample was stratified into three data-driven subgroups based on the CPP predictor variable using the aforementioned tertile cut-off points. Prior to conducting the one-way analysis of variance (ANOVA), fundamental statistical assumptions were evaluated. The assumption of normality was deemed acceptable based on skewness and kurtosis indices, and Levene’s test confirmed the homogeneity of variances across all subgroups. To quantify the magnitude of the subgroup differences and evaluate practical significance, partial eta squared ( $\eta_{p}^{2}$ ) was calculated alongside the F-statistics.

As shown in Table 8, compared with the low-perception group, both the moderate- and high-perception groups reported higher mean scores on student well-being, collaborative engagement, intercultural learning, student engagement, and inclusive learning climate. Furthermore, Games-Howell post-hoc tests were conducted for robust pairwise comparisons, as they do not assume equal variances. The post-hoc results confirmed that the mean differences among all three pairs (high vs. moderate, high vs. low, and moderate vs. low) were statistically significant ( p<0.05 ) across every indicator. Thus, the high-perception group showed the highest scores across all indicators, followed by the moderate-perception group, while the low-perception group showed the lowest levels. These results are consistent with the structural model but should be interpreted cautiously as descriptive subgroup patterns rather than evidence that CPP subgroups independently caused outcome differences.

**Table 8:** Subgroup comparisons across perceived CPP levels.

Outcome variable	Low group (*n* = 109) mean ± SD	Moderate group (*n* = 149) mean ± SD	High group (*n* = 115) mean ± SD	*F*	*p*	$\eta_{p}^{2}$
Student well-being	3.39 ± 0.52	3.73 ± 0.49	4.08 ± 0.46	46.27	<0.001	0.200
Collaborative engagement	3.46 ± 0.50	3.82 ± 0.47	4.11 ± 0.44	52.84	<0.001	0.222
Intercultural learning	3.31 ± 0.55	3.69 ± 0.51	4.03 ± 0.49	41.95	<0.001	0.185
Student engagement	3.41 ± 0.49	3.80 ± 0.45	4.15 ± 0.43	58.73	<0.001	0.241
Inclusive learning climate	3.52 ± 0.47	3.90 ± 0.44	4.22 ± 0.41	61.18	<0.001	0.249

Games-Howell post-hoc tests indicated significant differences for high versus moderate, high versus low, and moderate versus low CPP groups for every outcome (all *p* < 0.05).

### 3.6 Distributional differences across perceived CPP levels

**Figure 4:**
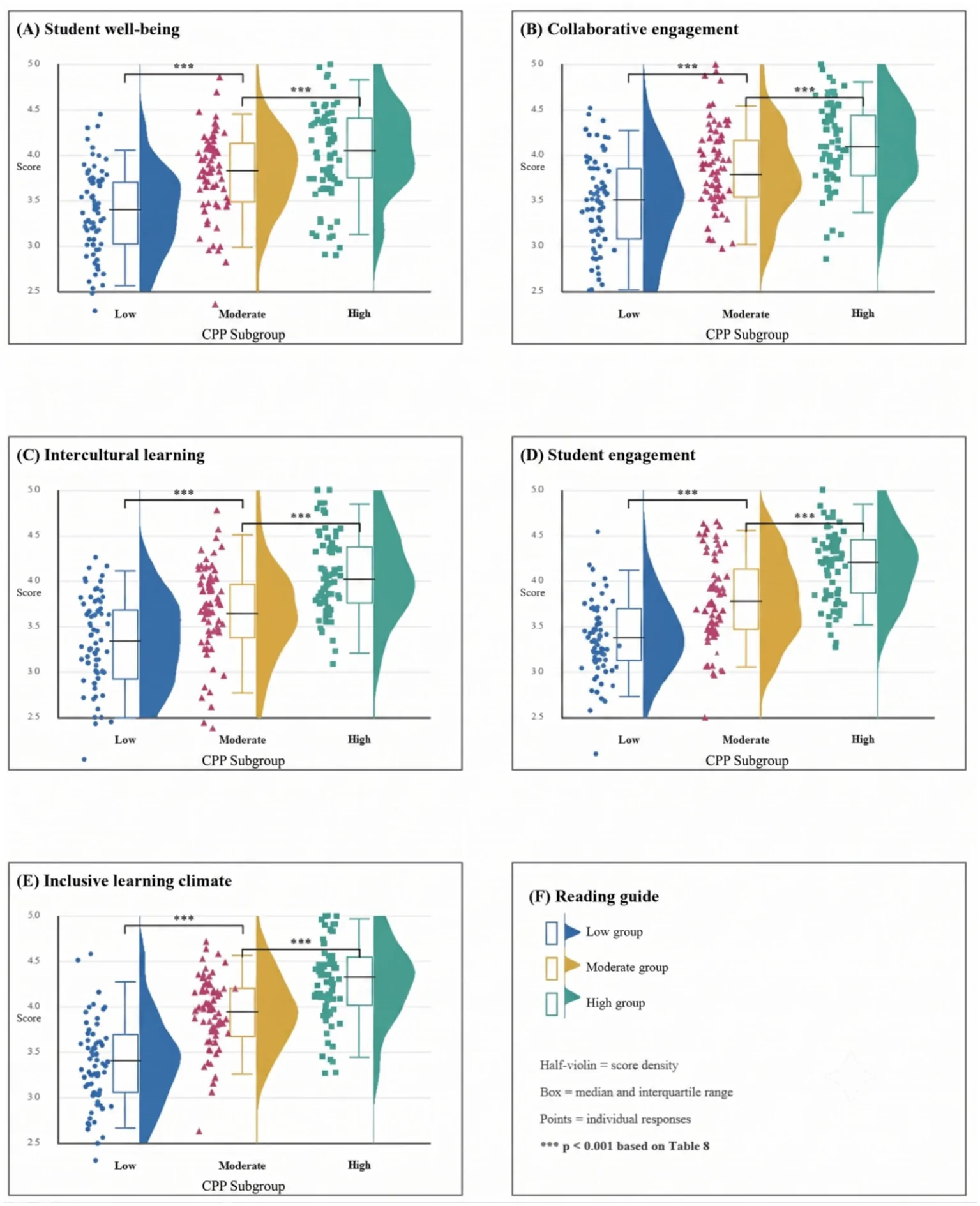
Raincloud plots of student outcomes and pathway variables across low, moderate, and high CPP subgroups. **(A)** Student well-being; **(B)** Collaborative engagement; **(C)** Intercultural learning; **(D)** Student engagement; **(E)** Inclusive learning climate; and **(F)** Reading guide for the half-violin density, boxplot, individual response points, and significance brackets. Colors and point shapes distinguish CPP subgroups, and *** indicates *p* < 0.001.

Figure 4 presents raincloud plots comparing low, moderate, and high CPP subgroups across student well-being, collaborative engagement, intercultural learning, student engagement, and inclusive learning climate. Each panel combines jittered response points, boxplots, and half-violin density displays, allowing the subgroup patterns reported in Table 8 to be inspected visually. These comparisons remain descriptive because the grouping variable is conceptually related to the main explanatory construct.

Across the five raincloud panels, students in the high-perception group generally showed higher distributions of well-being, collaborative engagement, intercultural learning, student engagement, and inclusive learning climate. The overlap among groups indicates that individual differences remained, but the overall gradient is consistent with the view that belonging to a higher CPP subgroup is linked to more positive psychological and interpersonal learning experiences.

Taken together, the visual results are consistent with the subgroup comparisons in Table 8. However, these distributional patterns should be understood as descriptive associations rather than evidence that creative and participatory learning design directly caused differences in student outcomes.

## 4 Discussion

This study examined six confirmatory hypotheses concerning direct and indirect associations between creative and participatory learning design (CPP) and students’ affective, collaborative, and intercultural outcomes. Statistical support was broad, but the coefficients were not uniform. CPP was more strongly associated with the proximal classroom perceptions of student engagement (*β* = 0.61) and inclusive learning climate (*β* = 0.64) than with the three developmental outcomes directly (*β* = 0.16–0.27). This layered pattern is compatible with an educational-interface account in which student and institutional factors meet in a psychosocial learning space (Kahu and Nelson, 2018). It does not, however, demonstrate that the variables unfolded in the proposed temporal order. The interviews provide contextual explanations for the statistical pattern, not independent proof of causal mechanisms.

### 4.1 Direct associations and support for H1–H3

H1 predicted positive direct associations between CPP and student well-being, collaborative engagement, and intercultural learning. The corresponding coefficients were 0.21, 0.27, and 0.16. Although these coefficients were not formally contrasted, their ordering is theoretically informative. Collaborative engagement is behaviorally closest to participatory tasks because both involve communication, shared responsibility, and joint problem-solving; well-being and intercultural learning are more distal experiences that may also depend on prior language competence, social relationships, and exposure to cultural difference. The present findings therefore add context-specific correlational evidence to prior studies of structured interaction and international collaborative learning (Arkoudis et al., 2013; Fukkink et al., 2024; Hackett et al., 2023; Lee et al., 2025; Shen and De La Garza, 2025). Because much of that literature concerns COIL, telecollaboration, or domestic-international student interaction, it should be treated as adjacent rather than directly equivalent evidence for co-located Sino-foreign programs.

H2 and H3 concerned associations with the proposed pathway variables. CPP was strongly associated with student engagement (*β* = 0.61) and inclusive learning climate (*β* = 0.64), while engagement was associated with inclusive climate (*β* = 0.29). These patterns align with accounts of engagement as an interaction between students and the educational environment rather than a fixed individual trait (Kahu and Nelson, 2018), and with cross-cultural evidence linking need-supportive teaching to well-being (Wang et al., 2021). They must nevertheless be interpreted cautiously. CPP included indicators of inclusive classroom interaction, whereas ILC assessed recognition, support, belonging, and willingness to participate; this construct-content proximity, combined with measurement by the same respondents at the same time, may partly contribute to the large CPP-ILC coefficient. The interviews further indicated that participation was not uniformly comfortable: linguistically imperfect contributions became easier only when students perceived error tolerance and social recognition. Similar variation has been reported in a Sino-American cooperative institute, where English proficiency, resource use, self-image, and engagement shaped students’ differential socialization (Zuo et al., 2024).

### 4.2 Student engagement, inclusive climate, and support for H4–H6

H4 and H5 were supported, but the outcome-specific coefficients add information beyond statistical significance. Engagement was most strongly associated with well-being (*β* = 0.39), followed by collaborative engagement (*β* = 0.23) and intercultural learning (*β* = 0.18). Inclusive climate was associated with well-being (*β* = 0.31), intercultural learning (*β* = 0.28), and collaborative engagement (*β* = 0.22). These point estimates are compatible with the interpretation that active involvement is closely aligned with students’ immediate emotional and learning experience, whereas recognition and belonging may be particularly relevant when students negotiate cultural difference. Research linking university belonging with motivation and enjoyment provides external support for this interpretation (Pedler et al., 2022). However, no formal tests compared the coefficients, and reciprocal explanations remain possible: students with greater well-being or intercultural confidence may also participate more actively and perceive the classroom more positively.

H6 concerned indirect associations through student engagement and inclusive learning climate. All six simple indirect estimates were significant, but their magnitudes differed descriptively. For well-being, the estimate through engagement was 0.238 and that through inclusive climate was 0.198. For intercultural learning, the estimate through inclusive climate was 0.179 compared with 0.110 through engagement, while the two estimates for collaborative engagement were almost identical (0.140 and 0.141). These differences were not formally contrasted and should not be described as statistically established differences between pathways. More importantly, the estimates are cross-sectional statistical decompositions rather than demonstrated temporal mediation. Cross-sectional mediation can yield biased estimates of longitudinal processes (Maxwell and Cole, 2007). The smaller sequential estimates through engagement and then inclusive climate (*β* = 0.039–0.055) are therefore best treated as hypothesis-generating patterns for longitudinal research, not evidence of a causal chain.

### 4.3 Practical implications and subgroup patterns

Mean scores increased across the low-, moderate-, and high-CPP groups for all five outcomes and pathway variables, with partial eta-squared values from 0.185 to 0.249. Because the groups were created from the CPP predictor, these differences visualize the continuous associations in a practitioner-accessible form but do not provide independent confirmation of the structural model. They also do not show that moving a student from one CPP category to another would cause an outcome change. As testable design considerations, instructors might combine open-ended tasks with explicit role negotiation, multiple modes of linguistic participation, error-tolerant feedback, and reflective dialogue. This interpretation is consistent with evidence that cross-cultural interaction often requires deliberate pedagogical structuring rather than simple co-presence (Arkoudis et al., 2013), but the effectiveness of these practices in Sino-foreign programs requires intervention-based evaluation.

The present data do not directly test class size, faculty readiness, or program-level support. These factors should therefore be framed as implementation questions rather than conclusions of the study. Future research could examine whether the observed associations vary by program, disciplinary field, English proficiency, and prior intercultural experience, and whether faculty development or structured participation protocols strengthen inclusive interaction. Such analyses would help distinguish effects attributable to classroom design from differences that students bring to the educational setting.

### 4.4 Study limitations

Several limitations bound the interpretation of the findings. First, the cross-sectional design cannot establish temporal ordering or causal mediation; estimates obtained when the predictor, pathway variables, and outcomes are measured at one occasion may not recover longitudinal processes (Maxwell and Cole, 2007). Reciprocal relationships are plausible, including the possibility that well-being, language confidence, or intercultural openness influences engagement and perceptions of classroom design. Second, all quantitative constructs were reported by the same respondents. Harman’s test and full-collinearity VIFs did not indicate a dominant common factor, but these diagnostics cannot eliminate social desirability, consistency motifs, or shared-method covariance. The uniformly significant paths should therefore be evaluated through coefficient magnitudes, confidence intervals, and explained variance rather than *p*-values alone. Third, CPP included an inclusive-interaction dimension, while the ILC mediator assessed recognition, support, belonging, and willingness to participate. Although HTMT results supported empirical distinction, this content proximity may have strengthened their association. Fourth, the model did not adjust for English proficiency, prior intercultural experience, year, major, or program, even though prior work in Sino-foreign education indicates that language background, resource use, self-image, and engagement influence students’ socialization (Zuo et al., 2024). Unmeasured confounding therefore remains possible. Fifth, students were nested within four programs, but the single-level PLS-SEM did not model program-level dependence; measurement invariance was also not tested across demographic or disciplinary groups. Self-selection into transnational programs further limits generalizability beyond these four mainland Chinese settings. Finally, the interview sample was small and coded by one researcher. The qualitative material provides explanatory depth and identifies boundary conditions, but it is neither representative nor independent causal corroboration. Longitudinal, multi-source, and multilevel studies, followed by intervention or quasi-experimental evaluations, are needed to test how engagement, psychological safety, language confidence, and belonging change over time.

## 5 Conclusion

This mixed-methods study provides context-specific evidence that students who perceived more creative and participatory learning design also reported stronger engagement, a more inclusive learning climate, greater well-being, more collaborative engagement, and more intercultural learning. Engagement and inclusive climate statistically accounted for part of these contemporaneous associations, while the interviews suggested that language confidence, error tolerance, peer trust, and social recognition may qualify how participation is experienced. The findings do not demonstrate that participatory pedagogy caused changes in student outcomes or that the proposed pathways unfolded sequentially. Instead, they identify theoretically plausible, outcome-specific associations that warrant longitudinal and intervention-based testing in Sino-foreign higher education.

## Data Availability

The raw data supporting the conclusions of this article will be made available by the authors, without undue reservation.
