# Supplementary material for: Participatory learning design and student well-being in Sino-foreign higher education: engagement and inclusive climate as psychological pathways
Source: Front Psychol. 2026 Aug 26;17:1913298. doi: 10.3389/fpsyg.2026.1913298 (PMC13553965; doi:10.3389/fpsyg.2026.1913298)
Supplement: Supplementary file 1 [file Table_1.DOCX]

**Supplementary Material**

**Supplementary Table S1**

*Joint Display Integrating Quantitative Structural Paths with Qualitative Explanatory Themes*

| **Quantitative Pathway (SEM Results)** | **Quantitative Finding** | **Qualitative Theme & Explanatory Evidence** | **Nature of Integration** |
| --- | --- | --- | --- |
| CPP → SE / ILC | Strong positive direct associations ( = 0.61, 0.64) | Theme: Psychological safety in the third space. "When teachers use open tasks, I don't feel judged for my English. It makes me want to speak up." | Convergence: Qualitative narratives support the interpretation that inclusive design may facilitate behavioral participation. |
| SE / ILC → SWB | Significant positive mediation toward well-being | Theme: Multilingual-intercultural identity negotiation. "Being engaged is exhausting at first due to the language barrier, but once the group accepts my perspective, it feels deeply rewarding." | Refinement/Nuance: Participant accounts indicate that the association with well-being may involve initial cognitive and linguistic friction. |
| SE / ILC → CE | Significant positive mediation toward collaborative engagement | Theme: Collaborative synergy through creative tasks. "We had to divide the work based on who understood the local context versus who was better at academic writing." | Extension: Participant accounts suggest that an inclusive climate may involve explicit strategic role negotiation among peers. |

*Note. CPP = Creative and Participatory Pedagogies; SE = Student Engagement; ILC = Inclusive Learning Climate; SWB = Student Well-Being; CE = Collaborative Engagement. The table demonstrates how qualitative narratives converge with, refine, and extend the cross-sectional statistical patterns Qualitative findings provide explanatory context and do not independently establish causality.*

**Supplementary Figure S1**

Spearman correlation matrix and construct-score distributions for CPP, SE, ILC, SWB, CE, and IL. Panel A shows standardized construct-score distributions. Panel B shows pairwise scatterplots, diagonal density curves, and Spearman correlation coefficients; *** indicates p < 0.001.
